# Expression of *cassini*, a murine gamma-satellite sequence conserved in evolution, is regulated in normal and malignant hematopoietic cells

**DOI:** 10.1186/1471-2164-13-418

**Published:** 2012-08-23

**Authors:** Anna Arutyunyan, Sonia Stoddart, Sun-ju Yi, Fei Fei, Min Lim, John Groffen, Nora Heisterkamp

**Affiliations:** 1 Section of Molecular Carcinogenesis, Division of Hematology/Oncology and The Saban Research Institute of Children’s Hospital Los Angeles, Los Angeles, CA, 90027, USA; 2 Medical Oncology, University Medical Center Groningen, Hanzeplein 1, 9713, GZ, Groningen, The Netherlands; 3 Laboratory of Molecular Immunology, The Rockefeller University, New York, NY, 10065, USA; 4 Leukemia Research Program, Children’s Hospital Los Angeles, Los Angeles, CA, USA; 5 Leukemia and Lymphoma Program, Norris Comprehensive Cancer Center, University of Southern California, Los Angeles, CA, USA

**Keywords:** Murine γ-satellite DNA, Major mouse satellite, Pericentromeric, Acute lymphoblastic leukemia, MEFs, Nilotinib, Stress, Cytotoxic drugs

## Abstract

**Background:**

Acute lymphoblastic leukemia (ALL) cells treated with drugs can become drug-tolerant if co-cultured with protective stromal mouse embryonic fibroblasts (MEFs).

**Results:**

We performed transcriptional profiling on these stromal fibroblasts to investigate if they were affected by the presence of drug-treated ALL cells. These mitotically inactivated MEFs showed few changes in gene expression, but a family of sequences of which transcription is significantly increased was identified. A sequence related to this family, which we named *cassini*, was selected for further characterization. We found that *cassini* was highly upregulated in drug-treated ALL cells. Analysis of RNAs from different normal mouse tissues showed that *cassini* expression is highest in spleen and thymus, and can be further enhanced in these organs by exposure of mice to bacterial endotoxin. Heat shock, but not other types of stress, significantly induced the transcription of this locus in ALL cells. Transient overexpression of *cassini* in human 293 embryonic kidney cells did not increase the cytotoxic or cytostatic effects of chemotherapeutic drugs but provided some protection. Database searches revealed that sequences highly homologous to *cassini* are present in rodents, apicomplexans, flatworms and primates, indicating that they are conserved in evolution. Moreover, *CASSINI* RNA was induced in human ALL cells treated with vincristine. Surprisingly, *cassini* belongs to the previously reported murine family of γ-satellite/major satellite DNA sequences, which were not known to be present in other species.

**Conclusions:**

Our results show that the transcription of at least one member of these sequences is regulated, suggesting that this has a function in normal and transformed immune cells. Expression of these sequences may protect cells when they are exposed to specific stress stimuli.

## Background

The bone marrow microenvironment provides protection to acute lymphoblastic leukemia (ALL) cells against drug treatment and is a frequent site of leukemia relapse. *Ex vivo*, primary pro-B ALL cells do not proliferate long-term without the presence of stromal support [1]. We therefore developed an *ex vivo* co-culture model consisting of mouse leukemic pro-B lymphoblasts [2] grown with mitotically inactivated mouse embryonic fibroblasts (MEFs). This system provides a generic type of protection to the ALL cells as is evidenced by the emergence of drug resistant ALL cells within 2-4 weeks of treatment with a moderate dose of a therapeutic drug [3-8].

Some of the factors produced by stromal cells that provide protection to the ALL cells have been identified, and include stromally produced SDF1α [4,9,10]. However, it is unclear if the presence of drug-treated ALL cells affects the stromal fibroblasts. The current study was initiated to examine this using expression profiling on the irradiated MEFs. These experiments led to the identification of an evolutionarily conserved family of multi-copy sequences, of which transcription is increased in both the ALL cells and the irradiated stromal cells when ALL cells are subjected to drug treatment.

## Results

### Mitotically inactivated stromal cells upregulate expression of a cluster of loci on chromosome 9 when exposed to drug-treated ALL cells

To provide stromal support to ALL cells without problems associated with the presence of two types of proliferating cells, we standardly mitotically inactivate the stromal MEFs by irradiation. Although these cells no longer divide, they are able to provide support to the ALL cells under steady-state growth conditions and also when the ALL cells are challenged by therapeutic drug treatment.

The murine ALL cells used here express the Bcr/Abl oncogene and are sensitive to the Abl tyrosine kinase inhibitor nilotinib. Previous experiments using different concentrations of nilotinib showed that 16 nM of this drug eradicates large numbers of 8093 ALL cells, but allows cells to grow out that have become tolerant to this concentration of drug [3].

**Figure 1:**
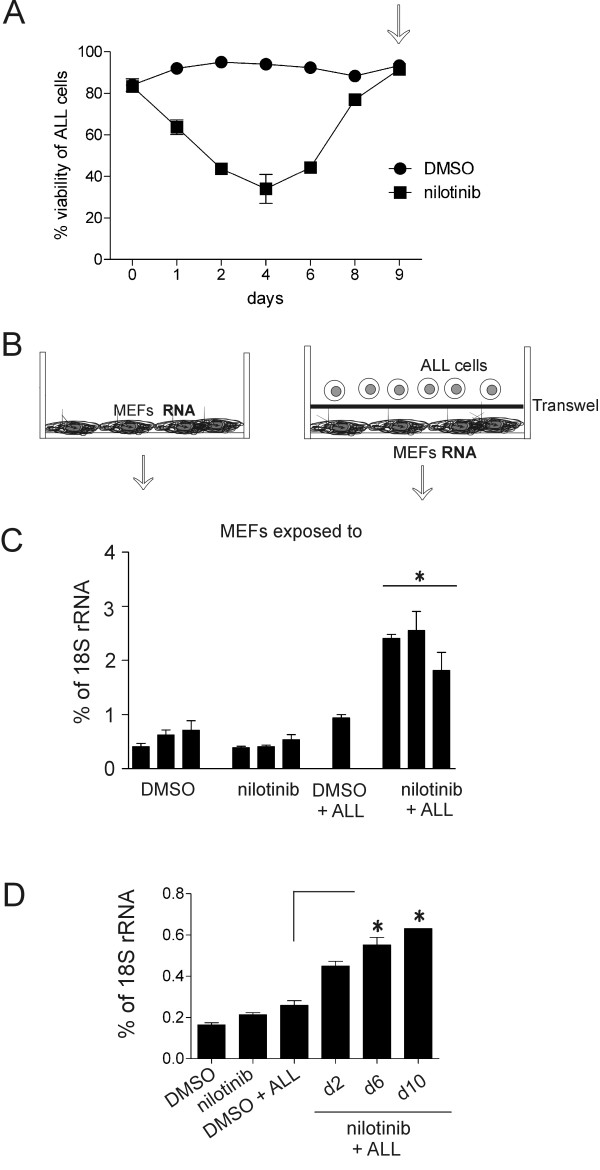
***Cassini *expression in MEFs in response to DMSO, nilotinib, or 8093 resistance to nilotinib.** (**A**) Viability of 8093 ALL cells over the course of a 9-day drug treatment with 16 nM nilotinib. MEFs were physically separated from the ALL cells by a Transwell. At day 9 (arrow), RNA was isolated from MEFs for gene expression profiling. Viability is expressed as total number of viable cells/total cell number. (**B**) Schematic indicating treatment of MEFs isolated for gene expression profiling. Left, irradiated MEFs were treated with DMSO or 16 nM nilotinib for 9 days. Right, irradiated MEFs were separated from 8093 ALL cells by a Transwell membrane. The co-cultures were treated with DMSO or 16 nM nilotinib for 9 days. (**C**) Confirmation of *cassini* expression using real-time RT/PCR on RNA isolated from MEFs. M, MEFs; D, DMSO; n, nilotinib (n = 3). Individual values for biological triplicates of samples M + D, M + n and M + ALL + n are indicated. Values are represented as mean ± SD of duplicate real-time RT/PCR. *p < 0.05 nil + ALL *versus* DMSO + ALL. (**D**) *Cassini* expression in MEFs treated with DMSO or 16 nM nilotinib, alone, or separated from overlying ALL cells by a Transwell membrane on the indicated days of drug treatment with 16 nM nilotinib or with DMSO (n = 1). *p < 0.05 d6 and d10 MEFs exposed to ALL/DMSO compared to MEFs exposed to ALL/nilotinib.

When these ALL cells are co-cultured with protective stromal cells, they adhere loosely to the top of this layer and migrate underneath it. To be able to isolate a pure population of protective MEFs, we therefore separated the ALL cells from the irradiated MEFs using Transwell membranes. Microarray analysis was performed on RNA isolated from MEFs exposed to DMSO, to nilotinib, and to nilotinib plus ALL cells at the end of the treatment on day 9 when the ALL cells had recovered (Figure 1A, B). As expected, there were minimal differences in the transcriptomes between MEFs treated with DMSO and nilotinib since this drug is a specific inhibitor of the deregulated Bcr/Abl tyrosine kinase oncogene in the lymphoblasts. Compared to DMSO-treated MEFs, only 59 probesets reported larger than 2-fold increased expression in MEFs exposed to nilotinib-treated ALL cells. Of these, only 13 reported values that exceeded a 3-fold upregulation.

Among those sets with highly increased expression, an interesting profile was reported by 8 probesets representing three genes located on chromosome 9 band A1 and one on chromosome 2 in band E1 (Additional file 1: Figure S1A, B). Expression was increased 3-10-fold in MEFs that were in contact with nilotinib-treated ALL cells (Additional file 1: Figure S1A). These probe sets are not present on previous murine Affymetrix gene expression arrays.

### Chromosome 9 cluster contains members of an evolutionarily conserved family of sequences

The annotation provided for one of the probesets, Gm10718 (Additional file 1: Figure S1B), indicates that its deduced amino acid sequence is similar to that of the hypothetical Plasmodium protein XP_675578. This region on mouse chromosome 9 contained 11 other loci with significant homology to the original 3 located on chromosome 9. Alignment of the deduced amino acid sequences from computer-generated transcripts of these loci revealed a family of highly related sequences (Additional file 2: Figure S2).

Further database analysis showed the existence of numerous physical homologous cDNAs (conceptual translation of nine distinct cDNAs, see Additional file 3: Figure S3), which are distinct from the cluster of sequences on the chromosome 9 locus. We conclude that the mouse genome must contain a large family of homologous but distinct genes of which at least nine are transcribed into RNA.

Homologous sequences were present on every mouse chromosome with 2753 mouse blast hits on 7_04_2011 in Refseq_genomic, many in the wgs and in the EST database (for example in murine HSC, Cd11c + dendritic cells, unfertilized eggs, mesenchymal stem cells, osteoblasts, lacrimal gland). Moreover, these sequences are highly conserved in evolution. The malaria parasite *Plasmodium* also contains numerous sequences, in addition to the one represented by XP_675578, with a high degree of similarity to this family. In the non-human, non-mouse ESTs database, sequences from *Danio rerio*, *Pythium ultimum* and *Amphioxus Branchiostoma* were found. Also, *Culex quinquefasciatus* contains a conserved hypothetical protein with high homology (ED538491.1; http://www.ebi.ac.uk/ena/). Human ESTs include for example AX647823, AW262283, AW262311, AW262398, BI764521 and BG272890. Similar sequences were also detected in other species, such as the lancelet *Amphioxus Branchiostoma*, a primitive “living fossil” vertebrate, in the est_others database.

### *Cassini* expression is high in hematopoietic cell types and is upregulated by bacterial endotoxin *in vitro* and *in vivo*

**Figure 2:**
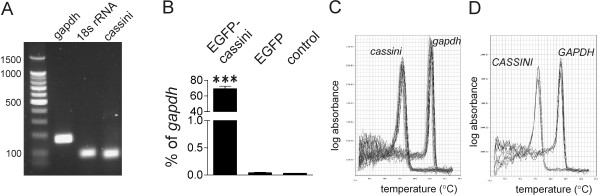
**Primer integrity and specificity analysis.** (**A**) 2% agarose gel electrophoresis of control 8093 RT/PCR products using *gapdh*, *18 s rRNA*, and *cassini* shows a single band for each target gene. (**B**) Real time RT/PCR on RNA isolated from non-transfected MEFs (control), MEFs transfected with an EGFP-*cassini* construct or with empty EGFP-C1 plasmid. Values (mean ± SD of triplicate real-time PCR) are expressed as percent of *gapdh*. (**C, D**) Derivative dissociation curves from real-time RT/PCR on mouse (**C**) and human (**D**) RNA confirm single product amplification. The *cassini* amplicon shows a single peak for both murine and human RNA with a T_m_ of ~76°C. *Gapdh* primers peak at T_m_ ~85.5°C. ***p < 0.001 EGFP-*cassini* compared to EGFP-C1 and control.

Because we could not verify the existence of cDNAs that matched the exact sequence of any members of the chromosome 9 cluster, we obtained a cDNA, AK089719, of which the deduced amino acid sequence was most homologous to the *Plasmodium* XP_675578 product (Additional file 3: Figure S3 and Additional file 4: Figure S4) to further investigate this family of genes. We named this specific gene product *cassini*. The cDNA was generated from RNA isolated from a murine activated spleen cDNA library. Primers for RT/PCR (indicated in the cDNA sequence in Additional file 4: Figure S4A) amplified a single product of the expected size (Figure 2A). We next subcloned the cDNA insert into an EGFP-expression vector, transfected this into MEFs and confirmed that the primer pair detected *cassini* RNA using real-time RT/PCR. As shown in Figure 2B, we detected high levels of the transcript in MEFs transfected with the cDNA compared to non-transfected cells or control cells transfected with EGFP. Dissociation curves of the product generated with this primer pair in real-time RT/PCR also support the amplification of a single product (Figure 2C, D).

**Figure 3:**
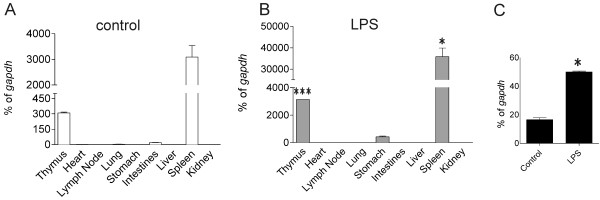
**Real time RT/PCR of whole organ *cassini *expression in control and LPS injected mice.** (**A**, **B**) *Cassini* expression in different murine tissues and 5 hrs after I.P. injection with 15 mg/kg *E. coli* LPS. Expression was verified twice in sets of control and LPS-treated mice (n = 2). Triplicate real-time values are represented as percent of *gapdh*. *p < 0.05 LPS spleen compared to control spleen. ***p < 0.001 LPS thymus compared to control thymus. (**C**) Real time RT/PCR on RNA from control and LPS (1 μg/mL) treated splenocytes isolated from a control mouse (n = 1).

Since AK089719 was isolated from hematopoietic tissue that had been activated, we first investigated *cassini* expression in mice exposed to bacterial endotoxin. As shown in Figure 3A, the highest levels of *cassini* RNA were present in the spleen and in the thymus of control mice. *E. coli* endotoxin treatment of the mice further induced the transcription of this locus 10-fold both in the thymus and spleen (Figure 3B). Transcription was also increased in isolated splenocytes stimulated *ex vivo* with LPS (Figure 3C).

### Acute lymphoblastic leukemia cells increase *cassini* transcript levels when exposed to therapeutic drugs

We next examined expression of *cassini* RNA in the MEF samples used for the gene expression profiling. These samples contain only MEF RNA since the fibroblasts were physically separated from the ALL cells by a membrane (Figure 1B). We found that nilotinib treatment of MEFs had little effect on *cassini* expression, whereas the presence of ALL cells treated with nilotinib induced a 2-fold increase (Figure 1C). To confirm that the presence of nilotinib-treated ALL cells specifically induced *cassini* in the MEFs, we isolated MEF samples on d2, d6 and d10 of nilotinib treatment. As shown in Figure 1D, there was a significant increase in expression of *cassini* in MEFs exposed to drug-treated leukemia cells compared to MEFs kept in the presence of DMSO-treated ALL cells.

**Figure 4:**
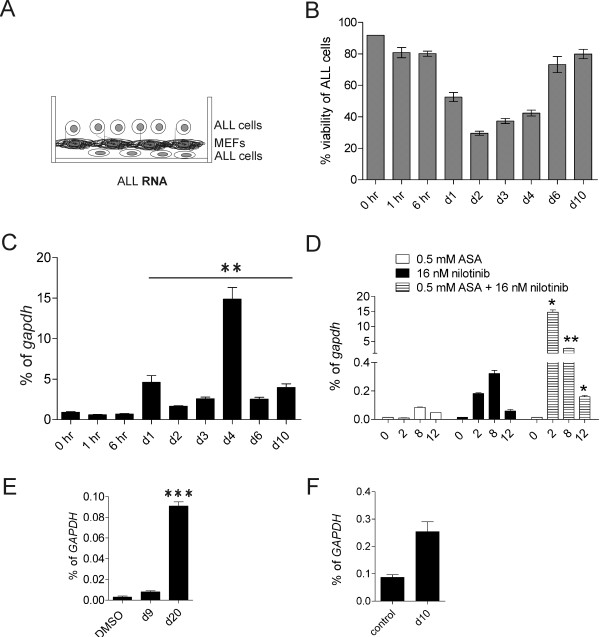
**Real time RT/PCR expression of *cassini *in pro-B ALL cells treated with therapeutic drugs in the presence of direct stromal support.** (**A**) Schematic representing the nature of a co-culture system in which ALL cells and stromal cells are in direct contact with one another. Due to partial infiltration of ALL cells underneath the stromal layer, only ALL cells in the supernatant were harvested for RNA isolation and gene expression purposes. (**B**) Viability of murine 8093 ALL cells over the course of drug treatment in the presence of MEFs. (**C**) Real time RT/PCR analysis of *cassini* expression in 8093 ALL cells at indicated time points during drug treatment (n = 3). **p < 0.01 d1-d10 compared to 0 hr. (**D**) 8093 cells treated with ASA, nilotinib or nilotinib + ASA (n = 2). ASA + nilotinib on d2, d8 and d12 *versus* nilotinib on d2, d8 and d12 *p < 0.05, **p < 0.01 and *p < 0.05, respectively. (**E, F**) Expression of *CASSINI* in human ALL cells in direct co-culture with irradiated OP9 stromal cells (n = 1 each) and treated with (**E**) 2.5 nM vincristine over the course of 20 days, with US7 cells harvested at indicated time points, day 9 and day 20 or (**F**) TXL2R cells cultured for 24 days without nilotinib (control), or cultured for 14 days without nilotinib, then treated with 500 nM nilotinib for 10 days. Values are represented as mean ± SD of duplicate real-time RT/PCR. ***p < 0.001 US7 d20 vincristine compared to DMSO; p = 0.06 (ns) TXLR control compared to TXLR2 d10 nilotinib.

To examine if ALL cells express *cassini* and whether or not drug treatment affects this, we isolated RNA from ALL cells treated with 16 nM nilotinib over the course of 10 days. After an initial drop in viability, this typically results in increased viability of the culture and emergence of ALL cells that are able to proliferate at that concentration of drug (Figure 4A, B). Figure 4C shows that levels of *cassini* were comparable to the 0 hr control at time points when viability of the ALL cells was still high. However, decreased viability correlated with induction of *cassini,* of which expression increased 5-15 fold. The peak of expression on day 4 correlated with the period of low viability before the culture started to recover. This suggests that *cassini* expression is related to a stress response of the ALL cells.

The combined treatment of the ALL cells with nilotinib and aspirin delays the emergence of drug-resistance (manuscript in preparation). To investigate the effect of this treatment on *cassini* expression, we treated mouse ALL cells co-cultured with irradiated MEFs with acetylsalicylic acid (ASA, aspirin), with nilotinib, and a combination of the two. As shown in Figure 4D, aspirin alone induced a modest upregulation of *cassini*. Interestingly, the dual treatment with nilotinib and aspirin caused a very high increase in *cassini* transcripts.

We additionally investigated if the mouse *cassini* primer pair would amplify a product in human RNA. Real-time RT/PCR on RNA isolated from control and vincristine-treated human ALL cells (Figure 4E) showed that human *CASSINI* RNA was present in these cells and that its expression was further increased by cytotoxic drug treatment. We also detected *CASSINI* RNA in TXL2R cells that had been made tolerant to 500 nM nilotinib (unpublished). Culture of these cells without drug for 14 days, followed by *de novo* treatment with nilotinib also induced a low increase in expression (Figure 4F). These results further confirm that *cassini* is evolutionarily conserved.

### Induction of *cassini* by specific stress

**Figure 5:**
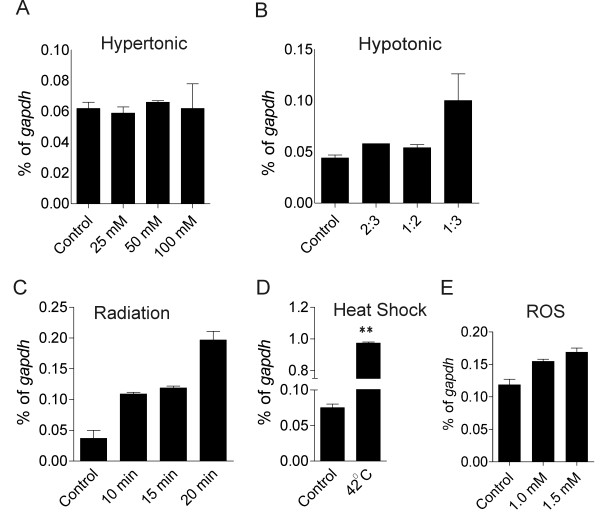
**Real time RT/PCR expression of *cassini *in response to cellular stress.** 8093 ALL cells were treated, as indicated in Methods, with (**A**) hypertonic medium (n = 1); (**B**) hypotonic medium (n = 3); (**C**) radiation (n = 2); (**D**) heat shock (n = 3); and (**E**) H_2_O_2_ (n = 3). Values are represented as mean ± SD of duplicate real-time RT/PCR. **D** **p < 0.01 heat shock versus control. Other comparisons, n.s., p > 0.05.

To further investigate if *cassini* upregulation in ALL cells correlates with cellular stress, we subjected 8093 pro-B leukemia cells to different types of treatments including temperature change, hypertonic and hypotonic stress, superoxide (H_2_O_2_) treatment and γ-irradiation. Hypertonic shock did not affect levels of *cassini*, and hypotonic treatment similarly had little effect (Figure 5A, B). Short-term radiation induced a non-significant increase (Figure 5C), and exposure to up to 1.5 mM of H_2_O_2_ did not significantly alter its expression (Figure 5E). However, exposure of cells to elevated temperature did induce a significant increase (Figure 5D).

## Discussion

### Mouse Î³-satellite is not homologous to human Î³-satellite, but human-mouse homologous sequences exist

Overall, absolute levels of *cassini* in cells and tissues that expressed it varied significantly, but were very high as compared to that of the reference gene *gapdh* or 18 s rRNA, and in some samples exceeded those of the housekeeping transcripts. Therefore, it was remarkable that this family had not been characterized. Further database searches revealed that *cassini* and sequences related to it belong to a class of DNA that has been collectively named γ-satellite DNA in the murine genome. Satellite DNA was named as such due to its buoyant density on CsCl gradients, differentiating it from that of the bulk of eukaryotic DNA. This is a consequence of its different overall base pair composition. When we evaluated the first DNA sequence reported for a mouse major satellite DNA from 1981, we found it to be a *cassini*-like sequence [11]. Vissel and Choo [12] coined the term γ-satellite DNA for these sequences to distinguish this mouse satellite DNA from other satellite families, such as the mouse “minor” satellite, the human centromeric N satellite and a new p-satellite family. However, the term γ-satellite, as proposed by Vissel and Choo, remains confusing in that it suggests relatedness between such sequences from different species. Mouse γ-satellites were regarded as non-conserved in evolution, and indeed we found that the sequences described as human γ-satellite DNA repeats bear no sequence homology to the mouse γ-satellite DNA (not shown). In addition, mouse γ-satellite DNA is reported to be pericentromeric, but we were unable to detect any homology between the *cassini*-type sequences and the different human pericentromeric repeats reported in Eymery et al. [13]. We did find numerous human *cassini*-like EST sequences and two non-assigned human genomic clones from chromosome 7 and 18. However, in contrast to mouse, none of these sequences have been assigned to a chromosomal sublocation and therefore it is unknown whether the human *CASSINI* loci are pericentromeric.

### Similar to some classes of human pericentromeric repetitive DNA, *cassini* mouse γ-satellite is transcriptionally regulated

Interestingly, as reviewed in Ugarkovic [14], some of the other satellite DNAs are also highly conserved. To the extent that the conservation of a sequence in evolution, such as that of an exon *versus* an intron, signifies an evolutionary constraint on divergence because of loss of function, this would imply that sequences such as *cassini* have a function. Vourc’h and Biamonti [15] reviewed possible functions of satellite DNA, in particular human pericentromeric satellite DNA, which has been more extensively studied. Although the human pericentromeric satellites show no homology to the mouse γ-satellite DNA, those located in the human 9q12 region show increased expression in HeLa cells that are heat shocked or stressed. Our results show that *cassini* expression is regulated by heat shock suggesting that the human pericentromeric satellites and cassini share inducibility.

### Mouse Î³-satellite could be part of one large transcriptional unit

Vissel and Choo [12] described the basic repeating unit of the mouse γ-satellite DNA to be 234 bp, and the majority of monomers were reported to be organized into largely uninterrupted arrays that vary from a minimum of 240 kb to greater than 2000 kb in length. However, an inspection of the only segment of the mouse genome wherein the organization of the γ-satellite is precisely reported shows that the 234 bp sequences are in fact organized as exons within typical genes. The chromosome 9 cluster contains 12 blocks of these sequences (Additional file 1: Figure S1A), organized in a tandem head-to-tail configuration in the same transcriptional orientation. The 12 blocks each have a possible intron-exon structure, with the γ-satellite sequences interspersed by non-γ-satellite DNA sequences. In fact, the automated DNA analysis software has annotated this region as containing genes.

It is currently unclear whether this area would be transcribed as 12 units or whether some of the units could be spliced together to form a larger transcript. Murine cDNAs corresponding in size to the predicted transcripts of these units are listed in the databases. The only indication that a very large spliced transcript could exist is the fact that the *Plasmodium* genome also contains homologous sequences, organized in a typical exon-intron structure; the largest virtual cDNA that was generated by automated annotation would be composed of 26 exons containing multiple “γ-satellite” units.

### *Cassini* shows some of the features of a typical gene

Whereas in earlier years repetitive DNA, including γ-satellite DNA, was regarded as functionless, in the course of twenty years, views on mouse γ-satellite DNA appear to have evolved [14]. One paper reported detecting a transcript in senescent cardiac muscle [16], and a different study linked mouse γ-satellite transcription to the cell cycle [17] suggesting that some of these sequences may be transcribed.

Our current study, using real time RT/PCR, is the first to extensively analyze and quantify transcription of one such sequence, which we have named after the celestial satellite *cassini*. The primer pair that we selected detects a single product in the databases, and the melting curves obtained from both mouse and human samples indicate the primers amplify a single product. However, we cannot definitively exclude the possibility that we are detecting transcripts from more than one locus because we presume that not all of these sequences are reported in the databases. Nonetheless, individual γ-satellite sequences that are reported as physical cDNAs have distinct nucleotide sequences. Thus, they represent distinct loci, and the *cassini* primers detect transcription from only a subset of these loci.

Furthermore, the detection of large differences in basal and induced transcription of *cassini* in different mouse tissues supports the specificity of its expression. If the *cassini* transcript is the product from a single gene, then specific stimuli, including endotoxin exposure and drug treatment of pro-B ALL cells, induce extremely high levels of this specific RNA. Our experiments standardized *cassini* levels to those of the abundant *gapdh* and, in some experiments, levels were measured that vastly exceeded those of *gapdh*. Because the highest basal levels of *cassini* were detected in thymus and spleen, its expression may be regulated in hematopoietic cell types, and we speculate that this could be promoted through transcription factors that were reported to bind mouse γ-satellite DNA including Ikaros, a B-lineage specific transcription factor that regulates the early development of hematopoiesis [18], Glfi1b, which is an important regulator of hematopoiesis [19-22] or YinYang1, which is needed for differentiation of proB to preB cells [23].

### Cassini protein?

It is currently unclear if *cassini* or other mouse γ-satellite RNA is translated into a protein (see Additional file 5: Figure S5 and Additional file 6) as we were unable to demonstrate the existence of a protein that corresponds in expression pattern to that of the *cassini* RNA. Although our data clearly show that a Cassini protein *can* be made, the product, if it exists, is likely to have unusual characteristics as is demonstrated by the migration abnormalities and aggregation of EGFP-Cassini after heat treatment in SDS-SB lysates (Additional file 5: Figure S5).

### Effect of *cassini* expression

Our experiments showed that induction of *cassini* RNA in cells correlates with specific stress stimuli, but our data do not provide information on whether the increased levels are detrimental to the cells or contribute a survival advantage. Because of the abundance of this RNA upon stress of cells, and the possibility that other transcribed loci exist in the genome with identical sequence, ablation of the RNA using siRNA does not appear technically feasible. We therefore used transient transfection in 293 cells to attempt to achieve expression levels that would significantly add to that already induced endogenously by stress, and also subjected the cells to a short-term treatment with chemotherapeutic drugs. The results of these experiments rule out the possibility that *cassini* induction correlates with or contributes to cell death, and suggest that high levels of this RNA provide some form of protection against the cytostatic activity of such drugs (Additional file 7: Figure S6).

## Conclusions

We conclude that γ-satellite repeat sequences are widely conserved and, as shown here, their transcription can be specifically regulated. Our analysis of *cassini* demonstrates that among this large family of loci, some are likely to participate in the response of acute lymphoblastic leukemia cells to cytotoxic drug treatment in cancer therapy in the presence of stromal support, and that this may provide some protection against these drugs.

## Methods

### Data access

The microarray data have been deposited in GEO under accession number GSE33329.

### Cell culture and experimental design

8093 cells, a murine pro–B ALL cell line that was isolated from a *BCR/ABL* transgenic mouse, human Ph-negative US7 and Ph-positive TXL2 cells have been previously described in detail [3,24]. Briefly, 8093 cells were cultured in McCoy’s 5A medium (Invitrogen) supplemented with 15% fetal bovine serum (FBS), 1% Glutamax, HEPES, 0.1% beta-mercaptoethanol, 1% penicillin/streptomycin, and 0.01 ng/mL of IL-3. US7 and TXL2R were cultured in αMEM (Invitrogen) supplemented with 20% FBS, 1% Glutamax, and 1% penicillin/streptomycin. TXL2R cells were derived from TXL2 by prolonged culture in the presence of nilotinib. Cells are able to proliferate normally in the presence of 500 nM of the drug. Primary total mouse splenocytes were incubated for 5 hrs in RPMI (Invitrogen) supplemented with 10% FBS, 1% Glutamax and 1 μg/mL of *E. coli* endotoxin or 1xPBS. For drug treatment experiments, murine and human ALL cells were co-cultured, directly or indirectly, in the presence of mitotically inactivated stromal MEF or OP9 feeder layers, respectively. For collection of leukemic cells in Figures 4 and 5A, a direct co-culture was used that results in cell-cell contact. Although ALL cells migrate underneath the stromal layer, they also proliferate abundantly in the supernatant. Because the MEFs are fully adhered to the plates, a pure population of leukemic cells is available for isolation (see schematic Figure 4A). In indirect co-cuture, leukemic cells are cultured in Transwells with 0.4 μM pore size, which precludes cell migration, but allows for free exchange of co-culture media. The Transwells are placed over the MEF stromal layer, allowing for a clean isolation of the MEFs (see schematic Figure 1B). Drugs including nilotinib, vincristine, and/or ASA, were refreshed every 48 hrs. Atttainment of resistance was measured by viability changes over the course of the experiments. Viability was assessed using Trypan blue exclusion. The cellular stress treatments (Figure 5) were performed in the absence of stroma, as they were short-term and therefore did not necessitate MEF support. Heat stress was applied for 30 minutes at 42°C in a water bath, with control cells maintained at 37°C under the same conditions. Cell viability in these experiments using Trypan blue exclusion was determined to be higher than 90%. ROS stress was induced by treating cells with the indicated concentrations of H_2_O_2_ for 3 hrs at 37°C. γ-irradiation was carried out on cells in suspension using a Gammacell 1000 (Nordion International, Inc). Cells were irradiated for the indicated duration at a dose rate of 2341 rads/4 min, followed by a 1 hr recovery at 37°C. Hyper-osmotic stress was generated by supplementing the McCoy’s 5A culture medium with 25–500 mM sorbitol. Cells were treated for 24 hrs at 37°C. Hypo-osmotic stress conditions were generated by diluting McCoy’s 5A medium with sterile, de-ionized water. Cells were incubated in the indicated dilutions for 1 hr at 37°C. Experiments involving transfection of EGFP-*cassini* into COS-1 and 293 FT HEK cells are described in more detail in Additional file 6.

### RNA purification from tissue and cells

All animal experiments were carried out in accordance with Institutional IACUC and NIH guidelines. For tissue expression analysis, mice were injected I.P. with 15 mg/kg E. coli LPS or saline. 5 hrs later, animals were sacrificed and tissue samples were harvested and stabilized in RNAlater (Qiagen) in preparation for RNA isolation using an RNeasy Plus Mini Kit and its specific protocol for RNA extraction from animal tissue. In addition to treatment with a gDNA removal column that is included in the kit, an on-column RNase-Free DNase (Qiagen) treatment was incorporated in the protocol to ensure complete removal of DNA. For *in vitro* experiments, cells were harvested and re-suspended in RNAprotect (Qiagen), and RNA was isolated as mentioned above. After assessment of quantity and quality of RNA using NanoDrop1000, purified RNA was reverse transcribed to cDNA using a High Capacity 1st Strand Synthesis kit (Applied Biosystems) in preparation for real time RT-PCR analysis.

### Real-time RT/PCR

Candidate primers in AK089719, which is 100% homologous to AK171984, were identified using NCBI Primer-BLAST and further analyzed for specificity using OligoCalc ( http://www.basic.northwestern.edu/biotools/oligocalc.html) and IDT ( http://www.idtdna.com/analyzer/Applications/OligoAnalyzer/Default.aspx). The selected primer pair did not yield any non-specific products and there were no homologous sequences in the RefSeqQ RNA database. No sequences in the Genome (all assemblies) and nr databases with 100% homology were found on 02/10/09. To further confirm primer specificity, PCR products were subjected to a melting curve analysis and 2% agarose gel electrophoresis analysis.

Primers for *cassini* target nts 882–905 (Upstream 5’-TGGCGAGGAAACCTGAAATGGTGG-3’) and 955–932 (Downstream 5’-TCGGTTTTCTTGCCGTATTCCACG-3’) of AK_089719 result in a 74-bp product. Murine glyceraldehyde 3-phosphate dehydrogenase was amplified using M*gapdh*U (5'-ACCCAGAAGACTGTGGATGG-3') and M*gapdh*D (5'-CACCACACACTTGATGGCCTGCAT-3'), which yielded a product of 171 bp. Murine 18 s rRNA was amplified using M18srRNAU (5’-GTGACTCTAGATAACCTCGG-3’) and M18srRNAD (5’-ACCATCGAAAGTTGATAGGG-3’), which yielded a product of 87 bp. Human glyceraldehyde 3-phosphate dehydrogenase was amplified using *HGAPDH*U (5’-CATTTCCTGGTATGACAACG-3’) and *HGAPDH*D (5’-GTCTCTCTCTTCCTCTTGTG-3’), which yielded a product of 128 bp. Real-time RT/PCR was performed as described in Behan et al. [25]. Briefly, reactions were carried out using 25 ng of cDNA, Power SYBR Green PCR Master Mix (Applied Biosystems), and 200 nmol/L primers in an ABI 7900HT Sequence Detection System (Perkin-Elmer, CA). *Cassini* transcript levels were normalized to *gapdh* or *18 s rRNA* and the SD determined from duplicate or triplicate wells.

### Gene expression profiling

Wild type MEFs were isolated using standard procedures. MEFs were plated on 3x 10-cm dishes to generate biological triplicate samples. When cultured with 8093 Bcr/Abl-positive ALL cells from a transgenic mouse model, they were separated by Transwell membranes (75 mm 0.4 μM pores). 8093 cells above the MEFs were treated with 16 nM nilotinib (n = 3). Two samples of MEFs co-cultured with ALL cells treated with DMSO were found to be contaminated by ALL cells and therefore a single value of ALL + DMSO-treated MEFs was used. MEFs were harvested on day 9. Control MEFs (n = 3) were treated with DMSO diluted 1:1000 or with nilotinib. RNA was isolated using TRIzol according to the manufacturer’s (Invitrogen Corporation, Carlsbad, USA) instructions. Microarray hybridization was performed by the University of Southern California Affymetrix MicroArray Core Facility at Children's Hospital Los Angeles. Briefly, RNA quality was first assessed using an Agilent Bioanalyzer (Agilent Technologies, Palo Alto, California, USA) and the 28S/18S ratios of all of the samples were between 1.3 and 2. RNA was converted to cDNA with Superscript Choice for cDNA Synthesis (Invitrogen, Carlsbad, California, USA) and subsequently converted to biotinylated cRNA with an Enzo High Yield RNA Transcript labeling kit (Enzo Diagnostics, Farmingdale, New York, USA). After hybridization to the murine Mouse Gene 1.0 ST arrays (28,000 transcripts, Affymetrix, Santa Clara, California, USA), the gene chips were automatically washed and stained with streptavidin-phycoerythrin using a fluidics system. The chips were scanned with a Hewlett-Packard GeneArray Scanner (Hewlett-Packard, Palo Alto, California, USA). Results were analyzed using Partek and Ingenuity Systems (version 7.1) software programs. Average microarray values from individual time points were calculated and log transformed.

### Database searches

Additional file 6 provides supplementary details on the database searches. In brief, to facilitate detection of homologous sequences, we translated the cDNA sequence of AK089719 into protein using ReBaseTools ( http://rebase.neb.com/rebase/) and then used its deduced amino acid sequence and that of XP_675578 and tblastn 2.2.21 and 2.2.25+ against htgs, nr, est, and est_others with the low complexity filter turned off.

## Abbreviations

ALL: Acute lymphoblastic leukemia; ASA: Acetylsalicylic acid; FBS: Fetal bovine serum; I.P.: Intraperitoneal; LPS: Lipopolysaccharide; MEFs: Mouse embryonic fibroblasts.

## Competing interests

The authors declare that there are no competing interests.

## Authors' contributions

Conceived and designed the experiments: AA, SS, SjY, FF, PG, NF, JG, NH. Performed the experiments: AA, SS, SjY, FF, ML, PG, NF. Analyzed the data: AA, SS, SjY, FF, PG, NF, JG, NH. Wrote the paper: AA, NH. All authors read and approved the final manuscript.

## Supplementary Material

Additional file 1 **Figure S1.**A cluster of genes near the telomere of mouse chromosome 9. Graphic respesentation of Affymetrix microarray results and table describing 40 kb region on mouse chromosome 9 band A1. Click here for file

Additional file 2 **Figure S2.**Deduced amino acid sequences of the cluster of Genbank-designated genes on mouse chromosome 9. Alignment of deduced amino acid sequences of chromosome 9 cluster. Click here for file

Additional file 3 **Figure S3.**Translation of different physical mouse cDNAs including AK089719 (*cassini*) with substantial homology to the mouse chromosome 9 cluster. The deduced amino acid sequence of mouse cDNAs are aligned. Click here for file

Additional file 4 **Figure S4.**Cassini protein. Translation of *cassini* cDNA, recurring amino acid motifs, and predicted secondary structure showing putative transmembrane regions. Click here for file

Additional file 5 **Figure S5.**Protein product of EGFP-tagged *cassini* cDNA. Detection of EGFP-tagged Cassini protein in transfected COS-1 cells using polyclonal antisera and EGFP fluorescence. Click here for file

Additional file 6 **Description of data, methods and references for Figures S1-S6.** Contains a description of the results shown in Figures S1-S6, the methods that were used to generate those data, and references relevant to this information. Click here for file

Additional file 7 **Figure S6.**Effect of *cassini* on survival of drug-treated cells. 293 HEK cells were transfected with control pEGFP-C1 or pEGFP-*cassini* and compared for viability and cell numbers after treatment for 24 hours with the chemotherapeutic drugs cisplatin, etoposide and doxorubicin. Click here for file
