# Supplementary material for: Expression of cassini, a murine gamma-satellite sequence conserved in evolution, is regulated in normal and malignant hematopoietic cells
Source: BMC Genomics. 2012 Aug 23;13:418. doi: 10.1186/1471-2164-13-418 (PMC3505476; doi:10.1186/1471-2164-13-418)
Supplement: Additional file 1 — Figure S1.A cluster of genes near the telomere of mouse chromosome 9. Graphic respesentation of Affymetrix microarray results and table describing 40 kb region on mouse chromosome 9 band A1. [file 1471-2164-13-418-S1.pdf]

Figure S1.

A.

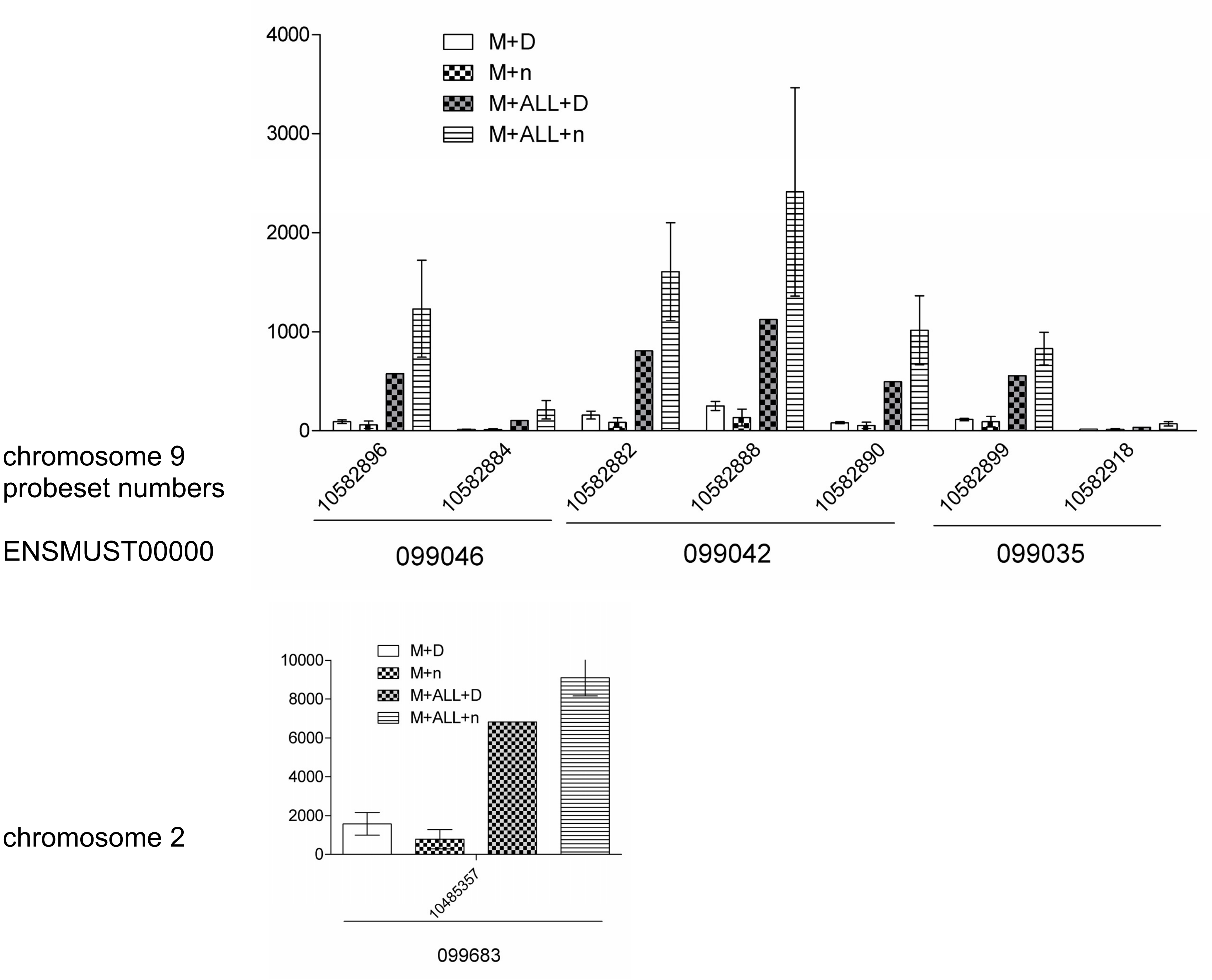
M+D
M+n
M+ALL+D
M+ALL+n
4000
3000
2000
1000
0
chromosome 9
probeset numbers
10582896
10582884
10582882
10582888
10582890
10582899
10582918
ENSMUST00000
099046
099042
099035
10000
8000
6000
4000
2000
0
chromosome 2
10485357
099683

B.

cassini-like cluster	[1]Genbank ID	[1]exons	[2]ENSMUST 00000	[3]ENSMUSP 00000	[1]strand	[4]Affy MoGene-1_0-st-v1 probeset ID	chromosome
**1**	Gm10722	4	151376	132394			9
**2**	Gm11168	4	115743	111408	fw		9
**3**	Gm10721	4	099051	096650	fw		9
**4**	Gm10720	[5]5	099050	096649	fw		9
**5**	Gm10719	4	099049	096648	fw		9
**6**	Gm11167	5	099047	096646	fw		9
**7**	Gm10718	4	[6]099046	096645	fw	10582896<br>10582884	9
**8**	Gm10717	3	075573	096644	fw		9
**9**	Gm10716	4	099042	096640	fw	10582882<br>10582888<br>10582890	9
**10**	Gm10106	3	099056	096655	fw		9
**11**	Gm17535	4	170073	132585	fw		9
**12**	[7]Gm10715	4	099035	096633	fw	10582899<br>10582918	9
	[8]Gm10800	5	099683	097275	rv	10485357	2

[1]Data from www.uswest.ensembl.org/Mus_musculus/Gene accessed 8/12/2011 (ENSEMBL Mus_musculus version 51.37d). These are the first annotated genes starting around 3 MB distal to the tip of band A1 chromosome 9 and span a total of around 40 kb.
[2]All RNAs are around 700 bp in length
[3]All are annotated as “known protein coding” and could encode around 225 amino acid residue polypeptides with a predicted 27 kDa MW. The putative polypeptides are indicated to contain up to 5 transmembrane helixes.
[4]Affymetrix probe set designation on MoGene-1_0-st-v1.
[5]This gene has a relatively standard exon-intron organization (69 bp coding- 410 bp intron-75 bp coding-234 bp intron, 292 bp coding-176 bp intron-117 bp coding-59 bp intron- 125 bp coding)
[6]Annotated by Affymetrix as “80% similarity with XP_675578”.
[7]A gene located immediately 3’ to Gm10715 is designated 4930433N12Rik and demarcates the end of the *cassini*-like cluster. 4930433N12Rik is annotated as generating non-coding RNA and is detected by Affymetrix probeset 10582916 (Suppl. Table 1; not included in Suppl. Fig. 1). There is no sequence similarity with the *cassini*-like cluster 1-12.
[8]Located on chromosome 2 band E1. This is non-pericentromeric.

Figure S1. A cluster of genes near the telomere of mouse chromosome 9. (*A*) Expression of the *cassini*-family gene cluster as measured by Affymetrix gene expression analysis. RMA-normalized, transformed Affymetrix gene array values from MEFs treated as indicated: M+D, irradiated MEFs treated with DMSO for 9 days; M+n, irradiated MEFs treated with 16 nM nilotinib for 9 days; M+ALL+D, irradiated MEFs separated by a Transwell from 8093 proB ALL cells treated with DMSO for 9 days; M+ALL+n, irradiated MEFs separated by a Transwell from 8093 proB ALL cells that had regained full viability after treatment for 9 days with 16 nM nilotinib (also see graph, Fig. 1*A*). Bars, SEM of biological triplicates. (*B*) The blocks of sequences 1-12 are listed from most telomeric to centromeric on chromosome 9.
