# Supplementary material for: Expression of cassini, a murine gamma-satellite sequence conserved in evolution, is regulated in normal and malignant hematopoietic cells
Source: BMC Genomics. 2012 Aug 23;13:418. doi: 10.1186/1471-2164-13-418 (PMC3505476; doi:10.1186/1471-2164-13-418)
Supplement: Additional file 2 — Figure S2.Deduced amino acid sequences of the cluster of Genbank-designated genes on mouse chromosome 9. Alignment of deduced amino acid sequences of chromosome 9 cluster. [file 1471-2164-13-418-S2.pdf]

Figure S2.

```
ENSMUSP00000                     PREDICTED PROTEIN
111408   MFLIFNDFHFSCHIPRPIVDIS--FPPFSVFFAIFHVPKCVFLIFRDFQFPRHIPGPTVCISHFSRFSVISSFFKSSSGCFSFSMIFSFLAIFH
096650             SCHIPCPTLDIS--FPPFSVFLAIFHVLKCVFLIFRDFQFSCHIPGPTVCISHFSRFSVILSFFKLSTACFSFSMIFSFLAIFH
096649   MFLIFNDFHFSCHIPCPTVDISK-FPPFSVFLAIFHVLKCVFLIFRDFQVSRHIPGPTVCISHFSRFSVILSFFKLSSGCFSFSLIFSFLAIFH
096648   MFLIFNDFHFSCHIPCPTVDISKFFPPFSVFLAIFHILKCVFLIFRDFQFSCHIPGPTVCISHFSRFSVISSFFKSSSGCFSFSMIFSFLAIFQ     94
096646   MFLIFHDFQFSCHIPCPTMDIS--FPTFSVFLAIFHVLKCVFLIFRDFQFSRHIPGPTVCISHFSGFSVISSFFKSSSGCFSFSMIFSFLAIFH
096645   MFLIFHDFQFSCHIPCPTVDIS--FPPFSVFLAIFHVLKCVFLIFRDFQFSCHIPGPTVCISHFSRFSLILSFFKLSTACFSFSMIFSFLAIFH
096644   MFLIFHDFQFSCHIPCPTVEICK-FPPFSVFLAIFHVLKYVFLIFRDFQFSRHIPGPTVCISHFSRFSVILSFFKFSSGCFSFSLIFSFLAIFH
096640    IFHISRFSFSCHIPCPTVDIS--FPPFSVFLAIFHVLKCVFLIFSDFLFSRHIPGPTVCISH-SRFSVISSFFKLSSGCFSFSMIFSFLAIFH
096655   MFLIFHDFQFSCHIPCPTVDIS--FPPFSVFLAIFHVLKCVFLIFRDFQFSCHIPGPTVCISHFSRFSVILSFFKLSSGCFSFSLIFTFLAIFH
0996633  MFLIFHDFQFSCHIPCPTVDIS--FPPFSVFLAIFHVLKCVFLIFRDFQFSRQIPGPTVCISHFSRFSVILSFFNLSTACFSLSMIFSFLAIFH
cassini  MFLIFHDFRFSCRIPRLTVNISK-FSTISGFLAIFHVLLCVFLIFRDFQFSRHIPGPSVCISHFSCFLVISSFFKSSSGCFSFSMIFSILAIFH

111408   VLQWTFLNFPPPFSVFLAIFHVLKCVFLIYRDFQFSRNIPGPTVCISHFFRFFMISSFFTLSSGCFSFSMIFTFLAIFHVLQWTFLNFPPFSVFS
096650   VLQWKFLNFPPPFSVFLAIFHVLKCVFLIFRDFQFSRHIPGPTVCISHFSGFSVISSFFKSSSGCFSFSMIFSFLAIFHVLQWTFLNFPPFSVFS
096649   VLQWTFLNFPPPFSVFLAIFHVLKCVFLIFSDFQFSRHIPGPTVCISHFFRFFMISSFFTLSSGCFSFSMIFTFLAIFHVLQWTFLNFPPFSVFL
096648   VLQWTFLNFPPPFSVFFAIFHVPKCVFLIFRDFQFPRNIPGPTVCISHFSRFSVISSFFKSSSGCFSFSMIFSFLAIFHVLQWTFLNFPPFSGFS    188
096646   VLQWTFLNFPPPFSVFLAIFHVLKCVFLIFSDFQFSRHIPGPTVCISHFSRFSVISSFFKLSSGCFSFSMIFSFLAIFHVPQWTFLNFPPFSSYS
096645   VLQWKFLNFPPPFSVFLAIFHVLKCVFLIFRDFQFSRHIPGPTVCISHFSRFSVISSFFKSSSGCFSFSMIFSFLAIFHVLQWTFLNFPPFSVFS
096644   VLQWTFLNFPPPFSVFLAIFHVLKCVFLIFSDFQFSRHIPGPTVCISHFSRFSVISSFFKVSSGCFSFSMIFSFLAIFHVLQWTFLNFPPFSGFS
096640   VLQWTFLNFPPPFSVFLAIFHVLKCVFLIFRDFQFSRHIPGPTVCISHFSGFSVISSFFKSSSGCFSFSMIFSFLAIFHVLQWTFLNFPTFSVFS
096655   VLQWTFLYFPPPFSVFLAIFHVLKCVFLIFRDFQVSRHIPGPTVCISHFSRFSVILSFFKLSSGCFSFSLIFTFLAIFHVLQWTFLNFPPFSVFL
0996633  VLQWTFLNFPPPFSVFLAIFHVLKCVFLIFRDFQFPRHIPGPTVCISHFSRFSVISSFFKTSSGCFSFFMIFSFLAIFPVLQWTFLNFPPFSVFL
cassini  VLQWIFLNFPPPFSVFLAIFHVLKCVFLIFRDFQFSRHIPGPSVCISHFSRFLVISSFFNSSSGCFSFSMIFSVLAIFHVLQWTFLNFPLFSVFL

111408     PYARSYRVHFSFFTFFSDFVIFHVVKWMFLIFNDFHFSC
096650     PYSRSYSVHFSFFTFFSDFVIFQVVKWMFLIFHDFQFSC
096649     PYSRSYSVHFSFFTFFSDFVIFQVVKWMFLIFHDFQFSR
096648     PYSRSYSVHFSFFTFFSDFVIFQVVKWLFLIFLDFQFSC      227
096646   FSPYSRSYSVHFSFFTFFSDFVIFQVVKWMFLIFLDFQFSC
096645     PYSRSYSVHFSFFTFFSDFDIFQVVKWMFLIFHDFQLSC
096644     PYSRSYSVHFSFFTFFSDFVIFQVFKWMFLIFLDFQFSC
096640     PYSRSYSVHFSFFTFFSDFVIFQVVKWMFLIFHDFQFSC
096655     PYSRSYSVHFSFFTFFSDFVIFQVVNCMFLIFYDFQFSC
0996633    PYSRSYSVHFSFFTFFSDFVIFQVVKWKFLIFHDFQLSS
cassini    AIFHVLKCVLLIFRDFQFSRHIPGPSVGISHFSRF*   stop codon
```

Figure S2. Deduced aminoacid sequences of the cluster of Genbank-designated genes on mouse chromosome 9 (ENSEMBL Mus_musculus version 51.37d). The deduced aminoacid sequence of *cassini* starting with the first ATG (methionine) in the cDNA is included for comparison. Not shown, ENSMUSP00000132394 and 00000132585.
