# Supplementary material for: Expression of cassini, a murine gamma-satellite sequence conserved in evolution, is regulated in normal and malignant hematopoietic cells
Source: BMC Genomics. 2012 Aug 23;13:418. doi: 10.1186/1471-2164-13-418 (PMC3505476; doi:10.1186/1471-2164-13-418)
Supplement: Additional file 3 — Figure S3.Translation of different physical mouse cDNAs including AK089719 (cassini) with substantial homology to the mouse chromosome 9 cluster. The deduced amino acid sequence of mouse cDNAs are aligned. [file 1471-2164-13-418-S3.pdf]

Figure S3.

```
XP_675578                                     FLPYSTSYIFLAIFHVLKCVFLIFRDFQFSRHNPGPSVCISHFSRFLVISSFFKSSIFLPYSTSYIFLAIFHVL
XP_675578                              KCVFLIFRDFQFSRHIPGPSVCISHFSRFLVISSFFKSSRGCFSFSMIFSFLAIFHVLHFLAIFQVLHFPRHISRPKVCISH


XP_675578 PYFHDFVIFQVVKWMFLIFHDFRFSCRIPRLTVNISKFSTISGFLAIFHVLLCVFLIFRDFQFSCHNPGPSVCISHFSRFLVISSFFKSSSGCFSFSMIFSFLAIFHVL
AK171928                    MIFSFLAIFHVLEWTFLNFPPFSVFLSIFHVLKCVFLIFHDFQFSRHIPGPSVCISHFSRFLVISSFFKSSSGCFSFSMILSFLAIFHVL
AK172170                    MIFSFLAIFHVLQWTFLNNPPFSVFLAIFHVLKCVFLIFRDFQFSCHIPGPSVCISHFSRFLVISSFFKSSSGCFSFSMIFSFLAIFHVL
AK172117                    MIFSFLAIFHVLEWTYINFPPFSIFLTIFHVLKCVFLIFRDFQFSRHIPGPSVCISHFSRILVISSFFKSSSGCFSFSMIFSYLAIFHVL
AK172311                    MIFSFLAIFHVLQGTFLNFPPFSVFLASFHVLKCVFLIFRDFQFSRHIPGFSVCISHFSRILVISSFFKSSCGYFSFSMIFSFLAIFLVL
AK156448               MFLIFHDFQFSCRIPCPTVDIPKFSTLFSFPCNISRPKVCISNFPRFSVFSPYTRSYSVHSHFSRFSVISSFFRSSSGCFSFSMIFSFLAIFHVL
AK156768               MFLIFHDFQFSCHISRPKVDISKYSTFSVFLAIFHVLKCVFLIFRDFQFSRHIPGPSVCISYFSRFLVISSFFKSSSGCFSFSIIFSFLVIFHIL
AK156257                MFLISMISVSCHIPRPTVDISKFPPFSVFLAIFHVLNCVFLIFRDFQFSRHIPGPSVCISHFSRFLVILSFFKASRGCFSFSMIFSFLAIFHVL
AK172626                                         HIPRPTVDISKFSTFFNFPRHIPGPSVCISHFSRFLVISSFFKSSSGCFSFSMIFSFLAIFHVL
cassini  FFTFFSDFVIFQVVKWMFLIFHDFRFSCRIPRLTVNISKFSTISGFLAIFHVLLCVFLIFRDFQFSRHIPGPSVCISHFSCFLVISSFFKSSSGCFSFSMIFSILAIFHVL


XP_675578QWTFLNFTPLSVFLAIFHVLQCVFLIFRDFQFSRHIPGPSVCLSHFSRFLVISSFFKSSRGCFSFSMIFNFLAIFLVLQWTFLNFPPFSVYLAILHVLNDFVIFQVVKWM
AK171928 QWTFLNFPPFSVFLDIFHVLKCVFLILRDFHFSRHIPGPSVCISHFSGFLVISSFFKSSSGCFSFSMIISFLAIFHVLQWTFLNFPPFSVFLDIFHVLKCVFLIFRDFQF
AK172170 QWTFLNIPPFSVFLTIFHVLKCVFLIFRDFQFSRYIPGPTVCISHFSRFSGISSFFKSSSGCFSFSMIFSFLAIFHVLQWTFLNFPPFSVFLAIFHVLNCVFLIIRDFQF
AK172117 QWTFLKFPLFSVFLAIFHVLKCVFLIFRYFQFSCHIPGPSVCISHFSRFLLISSFFKASSGCFSFSMIFSFLAIFHVLEWTFINFPPFSVFLAIFHVLKCVFLIFRDFKF
AK172311 QWTFLNFPPFSVFLAIFHVLKCVFLIFRDFQFSRHIPGFSVCISHFSRILVISSFFKSSSGCFSFSMIFSFLAIFQVLQCAFLIFHVF*
AK156448 QWTFLNFPLFSVFLAIFHVLNCVFLIFRDFQFSHHTPGPSVCISHFSRFSVISSFFKSSRGCFSFSMIFSFLAIFHVLQWTFLNFPPFSVFLAIFHVLKCVFLIFRDFQF
AK156768 QWTILNFPPFSVFLAIFHVLKCVFLIFRDFRFSRHIPGPTVCISHFSRFSVISSFYKWSSGIFSFSMIFSFLAIFHVLH*
AK156257 HWTFLNFPPFSVFLAIFHVLKCVFLIFRDFQFSRHIPGPSVCISHFSRILVFSSFFKSSSGCFSFSMIFSFLAIFHVLHWTFLNFPPFSVFSPYSRSFSGHFSFFTFFSD
AK172626 QWTFLNFPPFSVFLAIFHVLKCVFFIFRDFQFSPHIPGPSVGISHFSRFLVISSFFKSSSGCFSFSMIFSFLAIFHVLEWTFLNFPPFSVFLAIFHVLKCVFLIFRDFQL
cassini  QWIFLNFPPFSVFLAIFHVLKCVFLIFRDFQFSRHIPGPSVCISHFSRFLVISSFFNSSSGCFSFSMIFSVLAIFHVLQWTFLNFPLFSVFLAIFHVLKCVLLIFRDFQF


XP_675578 FLIFHDFQFSCHIPRPTVDISKFSNFFHFSSPFFMSISVYFSFSFVIFSFLAIFHVLQWTFLNFP*
AK171928  SRHIPGPSVCISHFSRFLVISSFFKSSSGCFSFSMIFSFLAIFHVLQWTFLNFPPFSVFLAIFHA……
AK172170  SRHIPGPSVCISHFSSFLVNSSFFKSSSVCFSFSMIFSFLAIFHV*
AK172117  SRHIPGLSVCISHFSRILVISSFFKSSSGCFSFSMIVS……
AK156448  SRHIPGPTMCISHFSRFSVILSFFMS*
AK156257  FVNFQVVKWMFLIFHDFQFSC….
AK172626  SRHIPGPSVCISHFSRFLVISSFFKSSSGCFSFSMIFRE….
cassini   SRHIPGPSVGISHFSRF*


XP_675578 PFSVFLAIFHVINWVFLIFRDFQFSRHIPGPSVCISHFSRFLVISSFFKSSSGCFSFSMIFSFLAIFHVLQWTLLNFPSFQFSLPYFTS
```

Figure S3. Translation of different physical mouse cDNAs including AK089719 (*cassini*) with substantial homology to the mouse chromosome 9 cluster. The deduced aminoacid sequence of the putative Plasmodium berghei ANKA partial gene product XP_675578 is aligned for comparison. Sequences ending with * have a defined stop codon. AK172170 also has a putative start codon defined by an in-frame upstream stop codon.
