# Supplementary material for: Expression of cassini, a murine gamma-satellite sequence conserved in evolution, is regulated in normal and malignant hematopoietic cells
Source: BMC Genomics. 2012 Aug 23;13:418. doi: 10.1186/1471-2164-13-418 (PMC3505476; doi:10.1186/1471-2164-13-418)
Supplement: Additional file 4 — Figure S4.Cassini protein. Translation of cassini cDNA, recurring amino acid motifs, and predicted secondary structure showing putative transmembrane regions. [file 1471-2164-13-418-S4.pdf]

Figure S4.

A.

```
 F  F  T  F  F  S  D  F  V  I  F  Q  V  V  K  W  M  F  L  I
PhePheThrPhePheSerAspPheValIlePheGlnValValLysTrpMetPheLeuIle   20
tttttcactttttttagtgatttcgtcattttttcaagtcgtcaagtggatgtttctcatt   60
 F  H  D  F  R  F  S  C  R  I  P  R  L  T  V  N  I  S  K  F
PheHisAspPheArgPheSerCysArgIleProArgLeuThrValAsnIleSerLysPhe   40
ttccatgatttttcggttttcttgccgtattccacgtcttacagtgaacatttctaaattt  120
 S  T  I  S  G  F  L  A  I  F  H  V  L  L  C  V  F  L  I  F
SerThrIleSerGlyPheLeuAlaIlePheHisValLeuLeuCysValPheLeuIlePhe   60
tccaccatttcaggtttcctcgccatatttcacgtcctattgtgtgtatttctcattttc  180
 R  D  F  Q  F  S  R  H  I  P  G  P  S  V  C  I  S  H  F  S
ArgAspPheGlnPheSerArgHisIleProGlyProSerValCysIleSerHisPheSer   80
cgtgattttcagttttctcgccatattccaggtccttcagtgtgcatttctcattttca  240
 C  F  L  V  I  S  S  F  F  K  S  S  S  G  C  F  S  F  S  M
CysPheLeuValIleSerSerPhePheLysSerSerSerGlyCysPheSerPheSerMet  100
tgtttttagtgatttcgtcattttttcaagtcgtcaagtggatgtttctcattttccatg  300
 I  F  S  I  L  A  I  F  H  V  L  Q  W  I  F  L  N  F  P  P
IlePheSerIleLeuAlaIlePheHisValLeuGlnTrpIlePheLeuAsnPheProPro  120
attttcagtattcttgccatattccacgtcctacagtggatatttctaaattttccacct  360
 F  S  V  F  L  A  I  F  H  V  L  K  C  V  F  L  I  F  R  D
PheSerValPheLeuAlaIlePheHisValLeuLysCysValPheLeuIlePheArgAsp  140
ttttcagttttcctcgccatatttcacgtcctaaagtgtgtatttctcattttcgcgat  420
 F  Q  F  S  R  H  I  P  G  P  S  V  C  I  S  H  F  S  R  F
PheGlnPheSerArgHisIleProGlyProSerValCysIleSerHisPheSerArgPhe  160
tttcagttttctcgccatattccaggtccttcagtgtgcatttctcattttcacgtttt  480
 L  V  I  S  S  F  F  N  S  S  S  G  C  F  S  F  S  M  I  F
LeuValIleSerSerPhePheAsnSerSerSerGlyCysPheSerPheSerMetIlePhe  180
ttagtgatttcgtcattttttcaactcgtcaagtggatgtttctcattttccatgatttt  540
 S  V  L  A  I  F  H  V  L  Q  W  T  F  L  N  F  P  L  F  S
SerValLeuAlaIlePheHisValLeuGlnTrpThrPheLeuAsnPheProLeuPheSer  200
agtgttcttgccatattccacgtcctacagtggacatttctaaattttccactttttca  600
 V  F  L  A  I  F  H  V  L  K  C  V  L  L  I  F  R  D  F  Q
ValPheLeuAlaIlePheHisValLeuLysCysValLeuLeuIlePheArgAspPheGln  220
gtttcctcgccatatttcacgtcctaaagtgtgtacttctcattttccgtgattttcag  660
 F  S  R  H  I  P  G  P  S  V  G  I  S  H  F  S  R  F*
PheSerArgHisIleProGlyProSerValGlyIleSerHisPheSerArgPhe **     238
ttttctcgccatattccaggtccttcagtgggcatttctcattttcacgtttttagtga
tttcgtcatttttcaagtcgtcaagtggatttttctcattttccattattttcagttttc
ttgccatattccatgtcctacagtggacatttctaaattttccacctttttcagttttcc
tcgccatatttcacgtcctaaagtgtgtatttctcattttccgtgattttcagttttctc
gccatattccaggtccttcagtgtgcatttctcattttcacgtttttagtgatttcgt
catttttaagtcgtcaagtggatgtttctcattttccatgattttcagttttcttgcca
tatttc
```

B.

```
   AA: MFLIFHDFRFSCRIPRLTVNISKFSTISGFLAIFHVLLCVFLIFRDFQFSRHIPGPSVCI
 Pred: -EEEEEEEEEEEE----EEEEEE-----EEEEEEEEEEEEEEEEE-----------EEEE
 Conf: 866830000111012454264123662111267831211688743120011154884265

Hconf: 000000000000000000000000000000000000000000000000000000000000
Econf: 099998248995732109999980001278999999799999999516743100009999
Cconf: 900001751004167890000019998721000000200000000483256799990000

   AA: SHFSCFLVISSFFKSSSGCFSFSMIFSILAIFHVLQWIFLNFPPFSVFLAIFHVLKCVFL
 Pred: EEEEEEEEE-EEEEEEEEEEEEEEEEEEEEEEEEEEEEEE-----EEEEEEEEEEEEEEE
 Conf: 756344211113333101016755312112342123232103763214676743422337

Hconf: 000000000000000000000000000000000000000000000000000000000000
Econf: 999999633999999647999999899899999999999850001999999999999999
Cconf: 000000366000000035200000010010000000000014999800000000000000

   AA: IFRDFQFSRHIPGPSVCISHFSRFLVISSFFNSSSGCFSFSMIFSVLAIFHVLQWTFLNF
 Pred: EE------------EEEEEE---EEEEEEE-------EEEEEEEEEEEEEEEEEEEE---
 Conf: 442531011145884155102311153110111133023542423146551023341126

Hconf: 000000000000000000000000000001000000000000000000000000000000
Econf: 991004620000009999961058999995234000699999999999999899999510
Cconf: 008994378999990000038941000003765999300000000000000100000489

   AA: PLFSVFLAIFHVLKCVLLIFRDFQFSRHIPGPSVGISHFSRF
 Pred: ---EEEEEEEEEEEEEEEEE-------------EEEEE----
 Conf: 851114575752643568440433125145882321311439

Hconf: 000000000000000000000000000000000000000000
Econf: 004999999999999999997000200000004999982000
Cconf: 995000000000000000002999799999995000017999

*   AA: Target sequence
* Pred: Predicted secondary structure (H=helix, E=strand, -=coil)
* Conf: Confidence (0=low, 9=high)
*Hconf: Confidence of helix  predictions
*Econf: Confidence of strand predictions
*Cconf: Confidence of coil  predictions
```

C.

```
   1-   43
       .............FFTF..FSDFV.IFQVVKWmFLIFHDFRFSCRIPRLTVNISKFS..........T.........I
  44-  101
       ....................SGFLAIFHVLLCvFLIFRDFQFSRHIPGPSVCISHFSCFLVISSFFKSSSGCFSFSMI
 102-  179
       FSILAIFHVLQWIFLNFPPFSVFLAIFHVLKCvFLIFRDFQFSRHIPGPSVCISHFSRFLVISSFFNSSSGCFSFSMI
 180-  238
       FSVLAIFHVLQWTFLNFPLFSVFLAIFHVLKCvLLIFRDFQFSRHIPGPSVGISHFSRF...................
```

D.

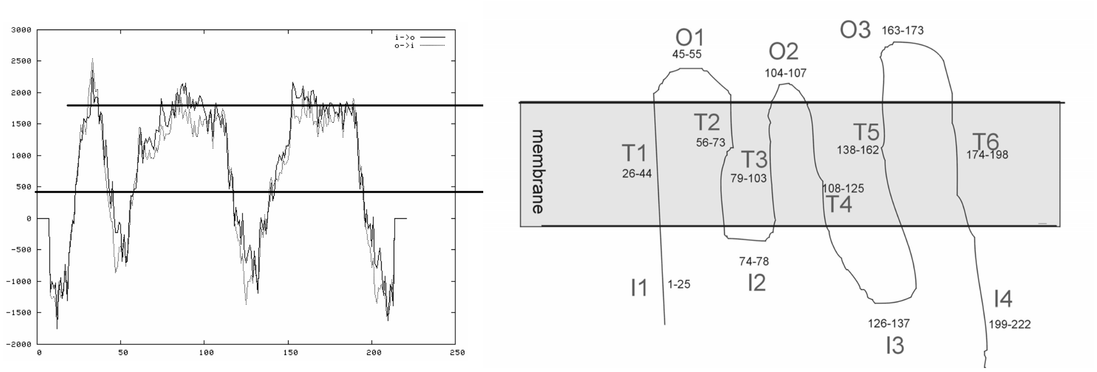

Figure S4. Cassini protein. (*A*) Deduced aminoacid sequence AK089719 *cassini.* The reverse compliment of the cDNA sequence of AK089719 was used for determining the deduced amino acid sequence. The reading frame is open 5’ to the first putative ATG (underlined). The sequences used as primers for real time RT/PCR are underlined in black and the peptide used to generate antisera in blue. (*B*) Recurring amino acid motifs within the putative Cassini protein. Blocks were identified manually and using RADAR (www.ebi.ac.uk/Tools/Radar/). (*C*) Predicted secondary structure putative Cassini protein. Secondary structure prediction was performed using YASPIN (http://www.ibi.vu.nl/programs/yaspinwww/). (Lin et al., 2005). (*D*) Schematic illustration putative Cassini transmembrane regions. TMPRED (*left*) and schematic (*right*) of putative transmembrane regions.
