# Supplementary material for: Expression of cassini, a murine gamma-satellite sequence conserved in evolution, is regulated in normal and malignant hematopoietic cells
Source: BMC Genomics. 2012 Aug 23;13:418. doi: 10.1186/1471-2164-13-418 (PMC3505476; doi:10.1186/1471-2164-13-418)
Supplement: Additional file 5 — Figure S5.Protein product of EGFP-tagged cassini cDNA. Detection of EGFP-tagged Cassini protein in transfected COS-1 cells using polyclonal antisera and EGFP fluorescence. [file 1471-2164-13-418-S5.pdf]

Figure S5

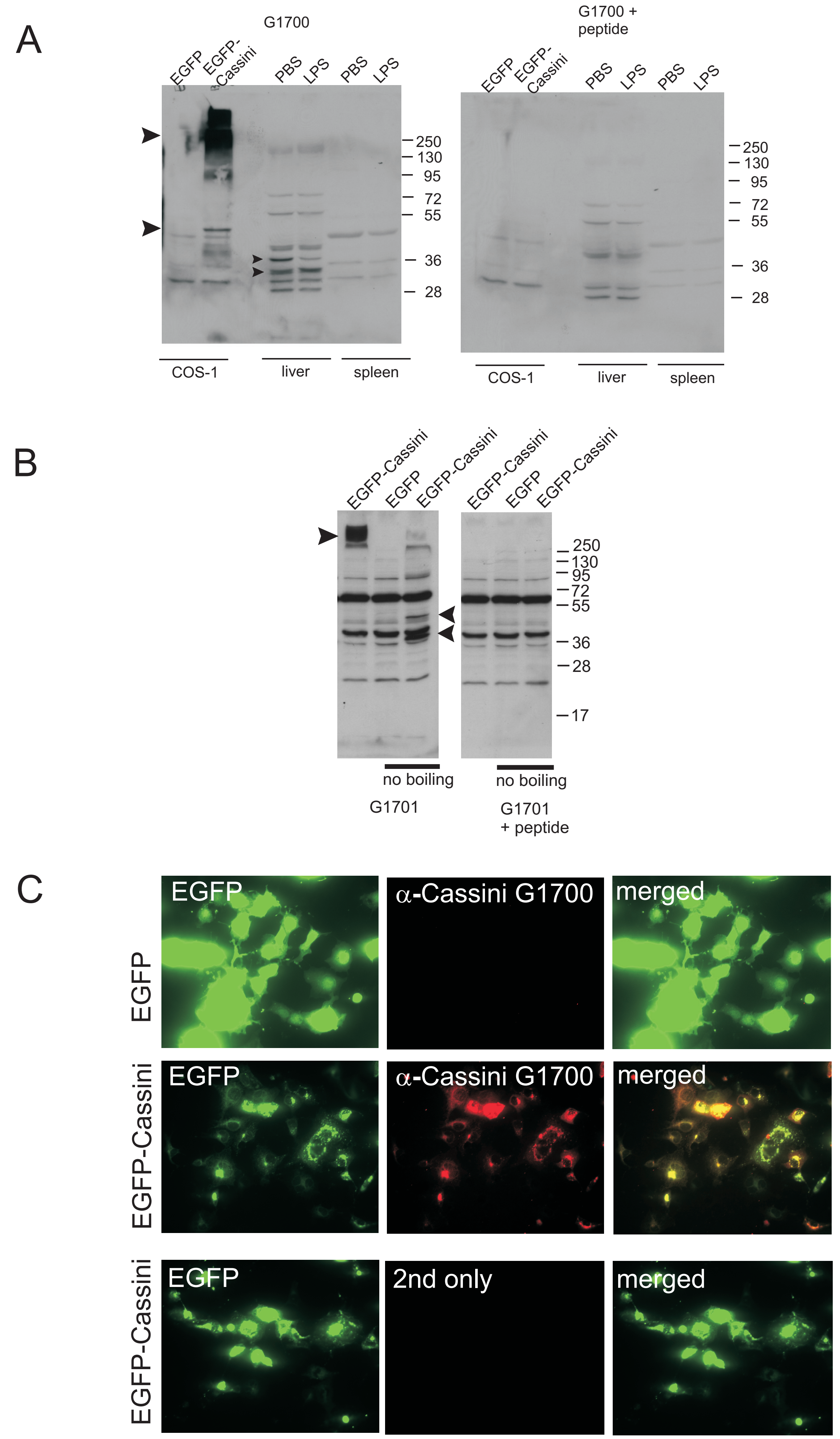
A
G1700
EGFP
EGFP-Cassini
PBS
LPS
PBS
LPS
250
130
95
72
55
36
28
COS-1
liver
spleen
G1700 + peptide
EGFP
EGFP-Cassini
PBS
LPS
PBS
LPS
250
130
95
72
55
36
28
COS-1
liver
spleen
B
EGFP-Cassini
EGFP
EGFP-Cassini
EGFP-Cassini
EGFP
EGFP-Cassini
250
130
95
72
55
36
28
17
no boiling
no boiling
G1701
G1701 + peptide
C
EGFP
α-Cassini G1700
merged
EGFP
EGFP
α-Cassini G1700
merged
EGFP-Cassini
EGFP
2nd only
merged
EGFP-Cassini

Figure S5. Protein product of EGFP-tagged *cassini* cDNA. (*A*) Western blot analysis of EGFP or EGFP-Cassini transfected into COS-1 cells (10 µg/lane) and lysates of liver and spleen (100 µg/lane) from mice injected with LPS or PBS as indicated. The left panel was incubated with affinity-purified polyclonal rabbit antisera (G1700) directed against a peptide from the putative C-terminal end of Cassini; samples in the right panel was preincubated with the peptide against which the antiserum was generated. Arrowheads in the left panel point to products that are specifically detected by the antibodies. (*B*) Samples from COS-1 transfected cells were treated as indicated below the panels. Arrowheads point to products specifically detected by the antibodies. (*C*) Detection of EGFP-Cassini in COS-1 cells. COS-1 cells transfected with EGFP or EGFP-Cassini encoding plasmids were evaluated for EGFP expression (*left panels*) or Cassini using polyclonal G1701 antisera as indicated. x20 magnification. *A*, *B*, the position of molecular weight standards are indicated to the right in each panel. Shown are representative results of experiments repeated at least two times.
