# Supplementary material for: Expression of cassini, a murine gamma-satellite sequence conserved in evolution, is regulated in normal and malignant hematopoietic cells
Source: BMC Genomics. 2012 Aug 23;13:418. doi: 10.1186/1471-2164-13-418 (PMC3505476; doi:10.1186/1471-2164-13-418)
Supplement: Additional file 6 — Description of data, methods and references for Figures S1-S6. Contains a description of the results shown in Figures S1-S6, the methods that were used to generate those data, and references relevant to this information. [file 1471-2164-13-418-S6.pdf]

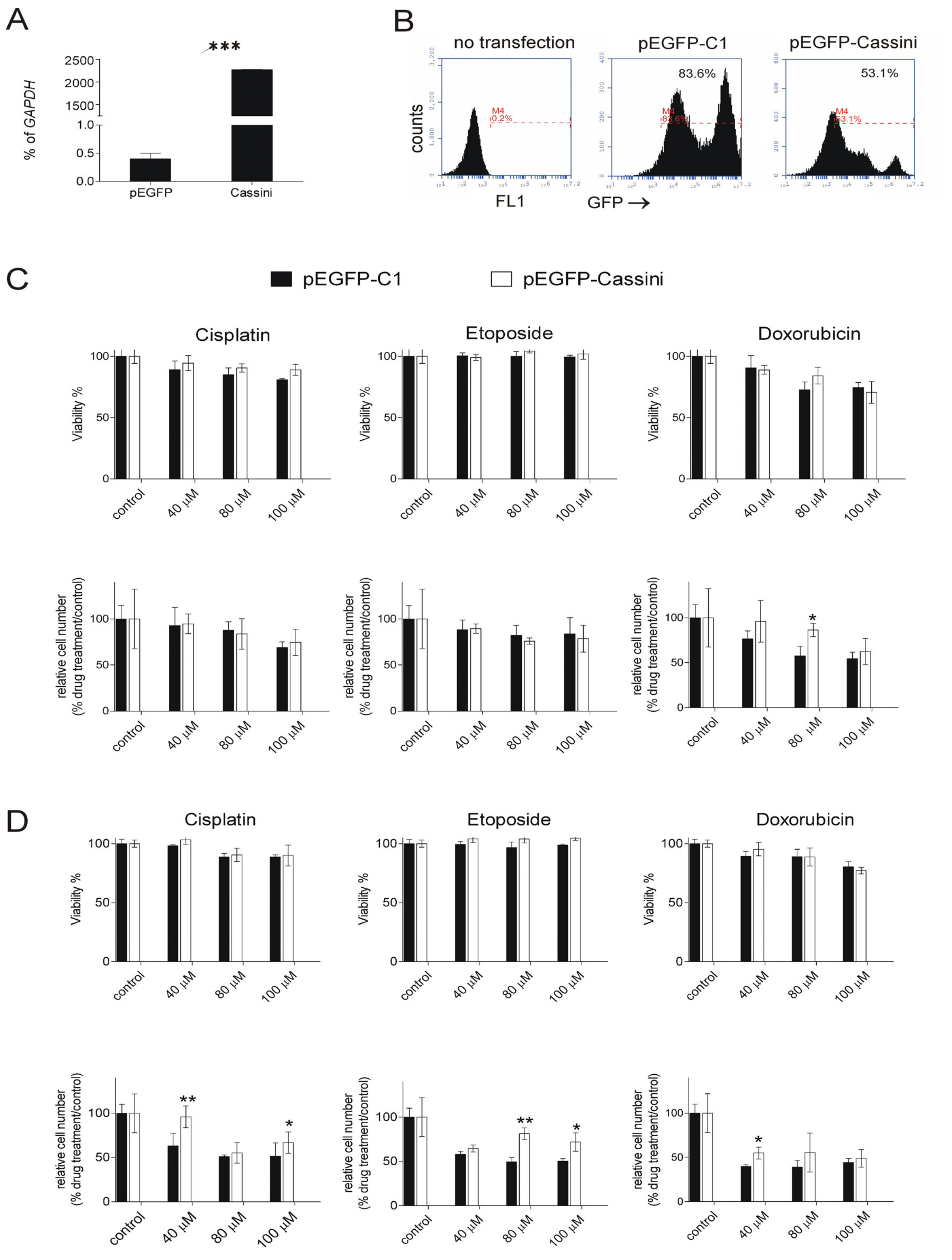

Figure S6. Effect of *cassini* on survival of drug-treated cells. Human 293 FT HEK cells transfected with pEGFP-C1 (expressing EGFP) or pEGFP-Cassini were assayed for (*A*), *cassini* RNA expression using real-time RT/PCR and (*B*) EGFP expression using FACS and (*C, D*) effect of *cassini* expression on cell growth, viability and chemotherapeutic drug treatment. *C* and *D* show independent transductions and experiments. All values represent mean ±SD of triplicate wells per treatment per transfectant. *p<0.05; **p<0.01; ***p<0.001.
