# Supplementary material for: Expression of cassini, a murine gamma-satellite sequence conserved in evolution, is regulated in normal and malignant hematopoietic cells
Source: BMC Genomics. 2012 Aug 23;13:418. doi: 10.1186/1471-2164-13-418 (PMC3505476; doi:10.1186/1471-2164-13-418)
Supplement: Additional file 7 — Figure S6.Effect of cassini on survival of drug-treated cells. 293 HEK cells were transfected with control pEGFP-C1 or pEGFP-cassini and compared for viability and cell numbers after treatment for 24 hours with the chemotherapeutic drugs cisplatin, etoposide and doxorubicin. [file 1471-2164-13-418-S7.pdf]

Supplementary information

Results

There were 8 related Affymetrix probe sets which reported increased transcriptional upregulation of 4 loci in irradiated MEFs that were communicating with drug-treated ALL cells (**Figure S1 A**). Seven of these loci originate from a region of 40 kb of DNA at 3 MB distal to the tip of 9. As indicated in **Figure S1 A** (#7, 9 and 12, shaded) this region contains 9 addional loci that are described in the database with different Genbank IDs (**Figure S1 B**). Each unit is listed as a possible transcriptional unit with an exon/intron structure and the same transcriptional orientation on the chromosome (for example ENSMUST00000099042, www.ensembl.org/Mus_musculus/Transcript/). The cluster on 40 kb would include a total of 48 coding exons.

The putative mRNAs have a corresponding hypothetical protein product associated with them (**Figure S1 B**) and each could produce an approximately 225 amino acid residue protein. Alignment of these (**Figure S2**) shows that they would be members of a family of closely related sequences. Further screening of database sequences for existing physical murine cDNAs identified many other homologous sequences (**Figure S3**; also AK089606, AK089889, AK089740, AK089559, AK089567, AK156805, AK156981, AK171891, AK172170). The hypothetical proteins generated from these transcripts are annotated as "phenylalanine-rich region profile containing proteins", that are exceptionally rich in phenylalanine (21%), serine (15%), isoleucine (11%), leucine (10%) and valine (8%). Around 27% of the residues are aromatic amino acids (H, F W, Y).

We used the deduced aminoacid sequence of *cassini,* tblastn (low complexity filter turned off) to identify closely homologous sequences in other organisms. Around 650 similar-sized (500 bp) human ESTs were identified, such as AW262311. One human sequence was found in the patent sequence data base (AX64782). A tblastn search against the human genome (accessed 8/2011; all assemblies and Celera WGSA) yielded no homologous sequences but the human HTGS and WGS databases contained sequences with a high degree of similarity such as AC083775 and AC090326 from chromosome 7 and 18. Human_sapiens *cassini*-like sequences could not be detected in RepBase (www.girinst.org).

Numerous homologous sequences were also found in *Plasmodium* subspecies that are infectious to mouse. The *Plasmodium* gene producing XP_675578, of which the sequence is partly aligned in **Figure S3**, is annotated as containing at least 8 exons and spanning around 2.6 kb. The putative protein product of this gene is predicted to encompass more than 529 amino acid residues and contain 13 probable transmembrane helixes (Q4YK64, www.uniprot.org). Numerous other genes in the *Plasmodium* genome encoding putative very large Cassini-like proteins include among others XP_679116, XP_678762, XP_679115, XP_679219 and XP_679129 with 1767, 1153, 1260, 1260 and 1689 amino acid residues. The largest is a 12 kb intron/exon genomic DNA fragment predicted to encode a 2335 amino acid polypeptide (Q4YA27) with 52 putative transmembrane regions encoded by a gene with at least 26 exons. The mosquito *Culex quinquefasciatus* also contains sequences with high similarity (EMBL eds38491.1 described a putative 365 amino acid integral to membrane protein).

We obtained a murine cDNA clone AK089719 isolated from a mouse activated spleen cDNA library from the RIKEN FANTOM collection (Yokohama city, Japan). The cDNA, which we named *cassini*, is predicted to encode a peptide of 222 amino acid residues (**Figure S4 A**). We analyzed its structure for helical transmembrane areas. 6 membrane helixes were predicted with a z score = 1.23 for the best model (**Figure S4 B, S4 C**).

The cDNA insert was subcloned into the eukaryotic expression vector pEGFPC1 to produce an in-frame fusion protein with EGFP. We also generated polyclonal rabbit antisera against a KLH-conjugated peptide encoded by the very C-terminal end the cDNA. Surprisingly, Western blotting of lysates from the EGFP-Cassini- transfected COS-1 cells on denaturing on SDS-PAA gels with these antibodies showed the presence of very high molecular weight proteins (>600 kDa) and minor bands, including one at around 48 kDa (**Figure S5 A** and **B**), whereas the predicted size of the product is around 54 kDa (EGFP 27 kDa + 27 kDa Cassini). The antibody reaction was specific, since addition of the peptide against which the antiserum was generated removed both the high molecular weight signal and the 48 kDa band (**Figure S5 A** and **B**).

Wasserman et al (2010) reported that the WDR62 protein exhibits a temperature-dependent mobility on SDS-PAA gels, depending upon whether or not the sample was boiled. We therefore compared lysates from EGFP-Cassini-transfected COS-1 cells with and without boiling. Remarkably, the high molecular weight material resolved into discrete bands when the sample was not boiled (**Figure S5 B**, lanes "no boiling").

Analysis of different mouse cells and tissues using real-time RT/PCR indicated that expression of *cassini* RNA is very high in spleen and low in liver and that moreover, endotoxin treatment of mice greatly increases the levels of RNA in spleen. However, Western blotting of lysates from the same animals using the antipeptide antisera (G1700, **Figure S5 A**; G1701, not shown) did not reveal the existence of a protein that was more abundant in spleen or of which expression increased upon treatment with LPS, either with or without boiling of the lysates. The antisera did detect proteins of around 38 and 32 kDa in liver (**Figure S5 A**; left panel, small arrowheads). No specifically induced product was detected in 8093 ALL cells treated with nilotinib.

We also examined the subcellular location of the EGFP-Cassini protein. As shown in **Figure S5 C**, the EGFP-*cassini* cDNA generated a protein that was detected in transfected COS-1 cells using both EGFP fluorescence and the G1700 antisera. The signal was punctate and clearly not nuclear, in contrast to the EGFP-only signal which was both nuclear and cytoplasmic (**Figure S5 C**). The EGFP-Cassinin did not co-localize with F-actin (results not shown). We conclude that *cassini* RNA can be translated into a protein but is currently not clear if such protein exists *in vivo*.

To investigate if the increased expression of *cassini* RNA contributes to death of stressed cells or provides protection, we transiently transfected pEGFP-C1 control plasmid or pEGFP-Cassini into 293 HEK cells. Real-time RT/PCR confirmed the high-level expression of *cassini* RNA in the pEGFP-Cassini transfectant (**Figure S6 A**) and FACS was used to compare transfection in both pEGFP-C1 control and pEGFP-Cassini (**Figure S6 B**). Viability of the cells transfected with EGFP or EGFP-Cassini was not significantly different (control samples **Figure S6 C** and **D**). 24 hours after transfection, we additionally treated some of the samples with different concentrations of cytotoxic chemotherapeutic drugs. We used cisplatin, a DNA cross-linking agent, the topoisomerase inhibitor etoposide and the anthracycline doxorubicin. These agents did not display a pronounced cytotoxic effect on 293 cells, as measured by the relatively small decrease in viability over 24 hours (**Figure S6 C, D**). All three drugs did exhibit a cytostatic effect, inhibiting cell proliferation. The proliferation of cells transfected with pEGFP-Cassini overall appeared to be less inhibited than that of cells transfected with pEGFP but this effect was not consistently noted in all samples (**Figure S6 C, D).** We conclude that increased levels of *cassini* do not promote cell death, and may be beneficial to cells experiencing extracellular stress.

Supplementary methods

Antisera against Cassini

Secondary structure of the putative protein product was analyzed using PHDhtm v1.96 (Profile fed neural network systems from HeiDelberg; Rost 1996; Rost et al., 1996) and YASPIN (http://www.ibi.vu.nl/programs/yaspinwww/).

The insert from clone AK08917 was released from the vector by digestion with BamHI and EcoRI and inserted in-frame with the EGFP gene into pEGFP-C1 (Clontech) digested with Bgl II x EcoRI. EGFP-Cassini and EGFP were transfected into COS-1 cells using Lipofectamine. We generated two polyclonal rabbit antisera (Open Biosystems, Huntsville, AL), G1700 and G1701, against a KLH-conjugated peptide RHIPGPSVGISHFSRF (**Figure S4**, underlined) encoded by the very C-terminal end the cDNA AK089719. G1700 was further affinity-purified using an Aminolink Plus Immobilzation kit (Thermo Scientific).

Cassini protein

EGFP-Cassini was transfected into COS-1 cells using Lipofectamine and Plus (Invitrogen). COS-1 lysates for Western blotting (10-20 µg/lane) were in SDS-SB or in modified RIPA buffer. Liver and spleen lysates (50-100 µg/lane) were made in SDS-SB or in 1% Triton lysis buffer. The different lysis buffers gave similar reuslts. G1701 antisera was diluted 1:500 for Western blotting and incubated overnight with membranes. For peptide competition experiments, 10-20 µl G1701 antiserum was pre-incubated with 100 µg of peptide in 1 ml 5% milk/TBST for 2 hrs at RT. Samples were run on 10% polyacrylamide gels. For immunocytochemistry, after fixation and permeabilization, slides were blocked with 1% BSA/PBS for 30 min. and incubated with affinity-purified G1700 for 2 hrs. Secondary antibodies were Cy3-anti-rabbit. Images were captured using an ImageLeica.

Treatment of transfected cells with drugs

293 FT HEK (human embryonic kidney) cells (0.3×10^6/well) were transfected with 0.5 µg pEGFP-C1 control or pEGFP-Cassini DNAs using Lipofectamine 2000 (Invitrogen). 24 hours after transfection, drugs were added. Different concentrations of doxorubicin, cisplatin, etoposide or DMSO (control) were used as indicated in the figure legend. After 48 hours, expression of the constructs was evaluated using FACS for EGFP (DMSO-treated only) and viable and total cell counts were performed using Trypan blue exclusion (all samples). The pEGFP-C1 control or pEGFP-Cassini-transfected cells treated with DMSO had different cell counts 48 hours after transfection. To be able to compare the effects of drug treatment on these different transfectants, therefore, we expressed cell numbers as the percentage of the drug treated cells/that of control DMSO treated cells.

Supplementary literature cited

Lin K, Simossis VA, Taylor WR, Heringa J: **A Simple and Fast Secondary Structure Prediction Algorithm using Hidden Neural Networks.** *Bioinformatics* 2005, **21:**152-159.

Rost B: **PHD: predicting one-dimensional protein structure by profile-based neural networks.** *Methods Enzymol* 1996, **266:** 525-539.

Rost B, Fariselli P, Casadio R: **Topology prediction for helical transmembrane proteins at 86% accuracy.** *Protein Sci.* 1996, **5:** 1704-1718.

Wasserman T, Katsenelson K, Daniliuc S, Hasin T, Choder M, Aronheim A: **A novel c-Jun N-terminal kinase (JNK)-binding protein WDR62 is recruited to stress granules and mediates a nonclassical JNK activation.** *Mol Biol Cell* 2010, **21:** 117-130.
